# Mechanistic insights into Rottlerin’s inhibition of MrkH-mediated biofilm and capsule formation in *Klebsiella pneumoniae*

**DOI:** 10.1186/s12866-025-04582-4

**Published:** 2025-12-27

**Authors:** Rosette S. Hanna, Mohamed Sebak, Ahmed M. Sayed, Ahmed O. El-Gendy, Mostafa N. Taha

**Affiliations:** 1 Department of Microbiology and Immunology, Faculty of Pharmacy, Nahda University, Beni-Suef, 62513 Egypt; 2 Department of Microbiology and Immunology, Faculty of Pharmacy, Beni-Suef University, Beni-Suef, 62514 Egypt; 3 Department of Pharmacognosy, Faculty of Pharmacy, Nahda University, Beni-Suef, 62513 Egypt; 4 Department of Pharmacognosy, College of Pharmacy, Almaaqal University, Basrah, 61014 Iraq

**Keywords:** Biofilm, MrkH, Rottlerin, *K. pneumoniae*, Molecular docking, Dynamics simulation

## Abstract

**Background:**

*Klebsiella pneumoniae* is a notoriously aggressive opportunistic pathogen within the *Enterobacteriaceae* family, with virulence factors, including polysaccharide capsules, lipopolysaccharide (LP>S), siderophores, and biofilm formation, serving as essential determinants of the pathogenicity. Biofilms in particular are associated with substantial nosocomial and community-acquired illnesses; moreover, the capsule enveloping *K. pneumoniae’*s surface further contributes to its viscous phenotype and virulence. This study explores the possible anti-virulence properties of the plant-derived compound Rottlerin using molecular docking aimed at a crucial protein implicated in biofilm formation in *Klebsiella pneumoniae*. Here, we investigate the molecular foundation of ligand-specific modulation of MrkH, a c-di-GMP-responsive transcriptional activator essential for biofilm development in *Klebsiella pneumoniae*. Utilizing a comprehensive methodology that encompasses molecular docking, dynamic modeling, and structural analysis, we evaluated the native c-di-GMP dimer–MrkH complex against the binding orientation and conformational impacts of the plant-derived chemical Rottlerin.

**Results:**

The sub-MIC of Rottlerin shows an inhibitory effect against some virulence factors, leading to a 57.6% decrease in biofilm formation, and a reduction in capsule size by 85.6% was observed; moreover, Rottlerin also significantly downregulated genes associated with these virulence factors. Through extensive molecular modeling (e.g., inverse docking, molecular dynamics simulation, and structural analysis), the c-di-GMP dimer was found to bind to rottlerin with a remarkable specificity, establishing stabilizing hydrogen bonds and distinctive π-cation interactions with Arg107 and Arg111, securing MrkH in an activation-ready configuration. Conversely, Rottlerin binds to the same pocket mainly via dual π-cation interactions with Arg107 and supplementary localized contacts; however, it is deficient in the extensive interaction network necessary for complete allosteric activation. Dynamic profiling by RMSF and PCA indicate that Rottlerin-bound MrkH exhibits an intermediate level of flexibility between the totally stable c-di-GMP-bound state and the highly dynamic apo form.

**Conclusion:**

These results substantiate the function of Rottlerin as a non-activating competitive binder, providing mechanistic insight into its potential as an anti-biofilm agent and building a foundation for the rational design of small-molecule inhibitors aimed at c-di-GMP regulatory pathways. Our findings demonstrate that Rottlerin is a potent and efficient sub-MIC inhibitor of *K. pneumoniae*’s ability to form biofilms and capsules.

**Supplementary Information:**

The online version contains supplementary material available at 10.1186/s12866-025-04582-4.

## Introduction

*Klebsiella* (*K*.) are nonmotile bacteria within the *Enterobacteriaceae* family, which are opportunistic pathogens commonly found in the flora of healthy individuals’ nasal passageways, throats, skin, and intestinal tract [1]. Despite their ubiquity, *Klebsiella* can still cause a range of infections, including pneumonia, soft tissue infections, surgical wound infections, urinary tract infections, infections in the bloodstream, and sepsis [2]. Notably, *K. pneumoniae* is a prevalent opportunistic pathogen acquired in hospitals, responsible for around 30% of all Gram-negative bacterial infections. Additionally, even with proper antibiotic treatment, the mortality rate for hospital-acquired pneumonia is over 50%. Both the occurrence and death rates of conditions linked to *K. pneumoniae* are notably high, especially among neonates, leukemia patients, and those with other immune deficiencies [3]. The extensive use of antibiotics has led to a significant increase in multidrug-resistant (MDR) *K. pneumoniae*, resulting in considerable challenges in its clinical management [4]. As a result, the World Health Organization (WHO) designates *K. pneumoniae* as a high-priority species and promotes the research and development of novel antibiotics to counteract the growing global challenge of antimicrobial resistance (World Health Organization, 2017) [5].

Virulence factors such as polysaccharide capsules, lipopolysaccharides (LPS), siderophores, and biofilm formation ability are crucial determinants of the pathogenicity of *Klebsiella* spp [6]. Biofilms are associated with significant nosocomial and community-acquired infections. They not only enable long-term colonization and avoidance of host defenses, but their formation also considerably increases antibiotic resistance, making infections more virulent and serious [7]. Furthermore, the capsule surrounding *K. pneumoniae*’s surface serves as a key virulence factor associated with its viscous phenotype and is hypothesized to protect against immune responses [8]. For example, *K. pneumoniae* effectively evades phagocytosis, complement activation, antimicrobial peptides, and specific antibodies by employing capsules that prevent bacterial adhesion [6].

These virulence mechanisms can be countered by naturally occurring compounds that display anti-virulent properties against *K. pneumoniae*. Some examples of these compounds include vitamin C [9], *Fructus mume* [10], and tea polyphenols [11]. Such agents have been shown to disrupt key virulence factors, which reduces the bacterium’s pathogenic potential. Nonetheless, numerous natural anti-virulence treatments continue to exhibit significant limitations. For instance, they must exist in relatively high concentrations to be useful, exhibit varying efficacy among various bacterial strains, and their methods of action remain partially understood. Such attributes highlight the constant demand for drugs that have broader, more consistent, and well-defined anti-virulence properties. These Data highlight the growing emphasis on targeting virulence rather than viability as a valuable and specific strategy for fighting infections.

In this regard, MrkH has gained attention as a critical target, given its central role in regulating biofilm formation and associated virulence in *K. pneumoniae*. The MrkH protein serves as a crucial transcriptional activator in the biofilm regulatory network of *K. pneumoniae*. It activates the *mrkABCDF* operon, which encodes type 3 fimbriae, and thereby promotes biofilm formation [12]. It can also positively autoregulate the transcription of the complete *MrkHI *operon, encompassing the gene for MrkI [12]. MrkI, in turn, has been implicated in the modulation of other cell surface components, including the polysaccharide capsule [13].

Thus, knocking out of MrkH directly hinders biofilm formation by diminishing the expression of essential mrk genes associated with Type 3 fimbriae development [14]. One study clearly states that the knockout of MrkH will likely hinder the bacterium’s capacity to colonize medical equipment and contribute to infections, hence diminishing its pathogenicity in clinical environments [13]. Similarly, another study showed that *ΔmrkH* mutants displayed a substantial decrease in biofilm formation on several medically critical materials when compared with the wild-type strain [12].

Recent efforts to explore MrkH included one investigation that demonstrated the inhibitory efficacy of Gallic acid (GA) against biofilms of *K. pneumoniae* [15]. Similarly, another study identified peptides, such as Neuropeptide B, that have a stronger binding affinity to MrkH than its native ligand, indicating their potential as inhibitors of *K. pneumoniae* biofilm development [16].

Given the urgent need for novel anti-virulence strategies, we explored our in-house natural product library using an inverse docking approach. Compounds were screened through PharmMapper, a pharmacophore-matching platform that links small molecules to potential protein targets derived from structural databases. Among all tested natural products, Rottlerin emerged as the only compound with a relevant fit score against MrkH, a c-di-GMP–dependent transcriptional activator that controls type-3 fimbriae expression and biofilm formation in *K. pneumoniae*. This unique hit suggested that Rottlerin might modulate MrkH-associated pathways, providing a rational basis for its selection as the focus of our computational and experimental investigations.

**Fig. 1:**
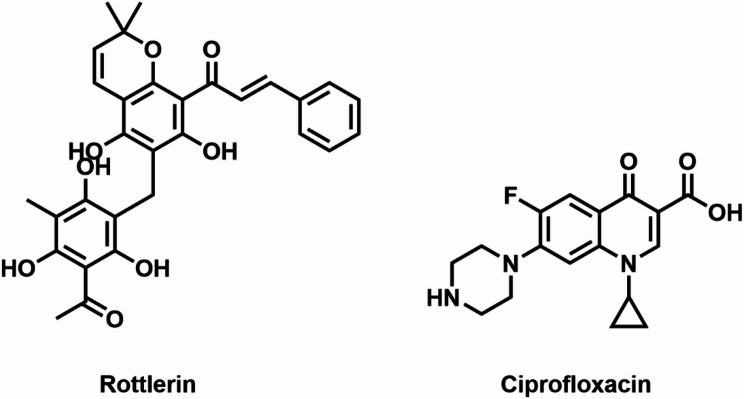
Chemical structure of Rottlerin and Ciprofloxacin

Accordingly, this study investigates the potential anti-virulence effects of Rottlerin (Fig. 1**)**, a polyphenolic natural compound derived from the Asian tree *Mallotus Philippensis* [17]. This investigation involves molecular docking targeting a key protein involved in biofilm formation in *K. pneumoniae* (ATCC700603). We aim to evaluate the possible impact of Rottlerin on virulence, focusing on genotypic alterations that influence virulence, a key factor in the persistence and resistance of *K. pneumoniae* infections.

## Materials and methods

### Screening for antimicrobial potential

A bacterial suspension of *K. pneumoniae* (ATCC700603) with an initial turbidity of 0.125 was added to LB agar plates. (0.5 McFarland, wavelength 600 nm). Subsequently, 100 µL of Rottlerin (a natural extract) and Ciprofloxacin (Fig. 1) (Arabco Med, Egypt), used as a positive control, were prepared at a concentration of 2 mg/mL and individually administered to each well on the LB plates and maintained at 37 °C for 24 h. The clearance zones were measured, and a zone of clearance that was more than 12 mm in diameter was regarded as active [18]. All assays were performed in three independent biological triplicates.

### Determination of the minimum inhibitory concentration (MIC), sub-MIC, growth monitoring, and viable bacterial count

The minimum inhibitory concentrations (MICs) of Rottlerin and Ciprofloxacin against *K. pneumoniae* were estimated using the broth microdilution method. In a plate with 96 wells, two-fold dilutions of Rottlerin and Ciprofloxacin were formulated in tryptone Soya Broth (TSB) (Oxoid, UK) with final concentrations spanning from 1.95 to 2000 µg/mL for Rottlerin and 1.36 to 1400 µg/mL for Ciprofloxacin. A 100 µL suspension of *K. pneumoniae* with a starting turbidity of 0.125 was introduced to each well and thereafter incubated at 37 °C for 24 h. The MIC was defined as the minimum concentration of Rottlerin and Ciprofloxacin that inhibited the growth of the *K. pneumoniae* strain (CLSI, 2020). MIC determinations and growth assays were conducted in three independent biological replicates.

To further evaluate bacterial growth dynamics, OD600 values were monitored over 24 h to confirm that Rottlerin at sub-MIC did not interfere with bacterial growth. In addition, a viable count assay was performed; Serial dilutions of the bacterial sample cultured with sub-MIC of Rottlerin and Ciprofloxacin were made in saline to reduce the concentration to a countable range. Each visible colony represents one viable bacterium or a colony-forming unit (CFU). Colonies are counted on plates, and the viable bacterial count (CFU/mL) in the original culture was calculated by multiplying the number of colonies by the corresponding dilution factor.

### Evaluation of biofilm formation using the crystal violet technique

The assessment of Rottlerin’s effect on biofilm formation was performed as previously described by [19] and [20] with some alterations. In summary, a *K. pneumoniae* suspension with an initial turbidity of 0.125 at 600 nm was used for all groups to ensure equal starting cell density for biofilm formation. The culture was then exposed to Rottlerin at its sub-MIC (0.375 mg/mL, ¼ MIC) and Ciprofloxacin at its sub-MIC (0.001 mg/mL, ½ MIC) for 24 h at 37 °C. Subsequently, sterile phosphate-buffered saline (PBS) was utilized to remove and eliminate the unattached bacteria. The biofilm was heat-fixed in an oven for 30 min at 60 °C, followed by staining with a 1% crystal violet solution for 15 min. The wells were then rinsed with water to remove any remaining residue stain. Crystal violet adhered to the biofilm was extracted using 96% ethanol. The contents were relocated to a new plate, and the absorbance of the crystal violet solution was quantified using a spectrophotometer at OD570. To serve as negative controls, medium-containing wells were implemented. A sub-MIC of Ciprofloxacin served as a positive control, as it has, in prior studies, been shown to impair biofilm production by *K. pneumoniae* [21]. The equation below was applied to assess the inhibition percentage [22]. Biofilm assays were performed in three independent biological replicates.

% inhibition = (Control OD - Test OD)/Control OD $\:\times\:$ 100.

### Transmission electron microscope (TEM)

Determination of the capsule size of *K. pneumoniae* was conducted through negative staining and visualized by TEM. Bacteria were incubated in LB broth with the addition of Rottlerin at its sub-MIC (0.375 mg/mL, ¼ MIC) and Ciprofloxacin at its sub-MIC (0.001 mg/mL, ½ MIC). TEM observation was carried out as previously described in [23], with minor modifications. 5 µl of bacterial culture were deposited onto a 200-mesh copper carbon grid for one minute. The grids were rinsed with a single drop of PBS, dyed with a single drop of 1% phosphotungstic acid (PTA) at pH 7.0, and subsequently dried by blotting on filter paper. Electron microscopy was carried out using a JEM-2100 electron microscope (JEOL, Tokyo, Japan). Capsular diameters were measured using GATAN software.

### Expression of virulence genes

The virulence gene expression of *K. pneumoniae* was measured through fluorescence real-time polymerase chain reaction (PCR), both with and without Rottlerin (0.375 mg/mL, ¼ MIC) and Ciprofloxacin (0.001 mg/mL, ½ MIC). Table 1 shows the primers utilized for the amplification of genes *mrkD*,* mrkA*,* fimH*,* luxS*,* treC*,* rmpA*,* magA*,* wbbM*,* mrkH*, and *K. pneumoniae 16–23 S ITS* (reference gene) [24, 25]. Prior to RNA extraction, bacterial cultures were standardized to the same optical density across all experimental groups to ensure equal starting cell numbers across groups. RNA extraction was conducted with an RNeasy Mini Kit (Qiagen). Reverse transcription of whole RNA was performed via RevertAid Reverse Transcriptase (Thermo Fisher, US). Real-time PCR was conducted utilizing SYBR Green Real-Time PCR Master Mix with a QuantiTect SYBR Green PCR kit (Qiagen, Germany). The reaction conditions for these genes are detailed in Table S1. *K. pneumoniae 16–23 S ITS* gene was used as a reference gene to standardize the quantitative PCR results and to determine the relative fold changes in gene expression.

**Table 1:** The primers utilized for the amplification of genes *mrkD*,* mrkA*,* fimH*,* luxS*,* treC*,* rmpA*,* magA*,* wbbM*,* mrkH*, and *K. pneumoniae 16–23 S ITS*

Gene	Primer Sequence (5’−3’)	Product Size	Reference
*fimH*	CCATCACCGCAGGATCGTTA	109	Wang et al., 2021 [25]
	ACCACGTCGTTATTGGCGTA		
*mrkA*	ACGTCTCTAACTGCCAGGC	115	
	TAGCCCTGTTGTTTGCTGGT		
*mrkD*	CCACCAACTATTCCCTCGAA	226	
	ATGGAACCCACATCGACATT		
*luxS*	AGTGATGCCGGAACGCGG	148	
	CGGTGTACCAATCAGGCTC		
*treC*	CCGACAGCGGGCAGTATT	71	
	CGCCGGATTCTCCCAGTT		
*rmpA*	TTCAGGGAAATGGGGAGGGTA	77	
	AAACGTCAAGCCACATCCATTG		
*magA*	GTCAGGCAGCTGTTGTGAAC	119	
	ACTTCTCGTATTTGCGGCGA		
*wbbM*	ATGCGGGTGAGAACAAACCA	122	
	AGCCGCTAACGACATCTGAC		
*mrkH*	GGAGTTGCGCAAACGTACTG	184	Designed in the current study
	TGTCAGGGCCGCATTAAACT		
*K. pneumoniae 16–23 S ITS*	ATTTGAAGAGGTTGCAAACGAT	130	Turton et al., 2010 [24]
	TTCACTCTGAAGTTTTCTTGTGTTC		

### Virtual target identification

The putative target characterization of Rottlerin was achieved via Pharmacophore-based virtual screening using PharmMapper [26]. This platform assigns a score to each molecule in the Protein Data Bank (PDB) that best fits a pharmacophore model that has been extracted and stored as a library of ligand datasets in mol2 format. To identify where a new molecule fits on the scale of all the pharmacophore scores, its fit score for each pharmacophore is determined, and each fit score is compared to the fit score matrix. The pure fit score that results from this procedure carries considerably more weight and assurance. The query structure was submitted to the platform in PDB format, and the retrieved results were exported as an Excel sheet, arranging the resulting protein targets according to their fit scores.

### Docking-based virtual screening

#### Ligand structure generation

OpenBabel software v.3.1.1 [27] was used to convert the structures’ SMILE codes to three-dimensional configurations that were subsequently subjected to a minimization of energy using the steepest descent technique with the same software. The minimization was performed using the force field MMFF94. Using AutoDockTools v.4.2, all torsions of the selected structures were assigned, and their Gasteiger charges were provided for all studied atoms in the structures [28].

#### Protein structure preparation

For docking screening, we used the crystal structure of *K. pneumonia* MrkH (PDB code: 5EJL) that has been described previously [29] and is available in the Protein Data Bank (10.2210/pdb5EJL/pdb). PDB fixer [30] was used to edit the downloaded structure, adding missing residues and atoms, and removing co-crystallized H_2_O and heteroatoms. Through AutoDock Tools v.4.2, polar hydrogen and Gasteiger charges were subsequently made available for both proteins.

#### Structural docking

The docking process was carried out using the PyRx platform’s built-in AutoDock Vina software [30, 31].

According to the co-crystalized ligands of both enzymes, the docking search grid boxes were determined to enclose them with a 20 Å^3^ total size perfectly.

The grid box’s coordinates were set to be x = −9.682; y = 4.274; z = −23.145. The level of exhaustion was held at 24. Pymol 3.1. software was used to evaluate and display docking poses. Exhaustion was set to 24. Ten poses were generated for each docking experiment. Docking poses were analyzed and visualized using Pymol 3.1. software [32].

#### Molecular dynamics simulation

MD simulations were performed using NAMD 3.0.0 [33, 34]., employing the CHARMM36 force field [35]. The protein systems were constructed using the QwikMD toolkit within VMD version 1.9.4 [36]. The initial configuration of the MrkH structure was prepared by adding missing hydrogen atoms, ensuring correct protonation states for physiological pH (7.4), and removing crystallographic water molecules. The system was solvated in an orthorhombic water box using the TIP3P water model, with a 25 Å buffer around the protein-ligand complex. Sodium and chloride ions were added to maintain electrostatic neutrality at a final ionic strength of 0.15 M.

Energy minimization was performed using a combination of steepest descent and conjugate gradient algorithms to remove steric clashes and relax the system. The system was equilibrated in two phases: (1) a 1-ns NVT equilibration to stabilize the temperature at 310 K using a Langevin thermostat and (2) a 5-ns NPT equilibration to stabilize pressure at 1 atm using the Berendsen barostat. During equilibration, positional restraints were applied to the heavy atoms of the protein and ligand to allow the solvent and ions to equilibrate around the system. Following equilibration, unrestrained production simulations were carried out. The parameters and topologies for the ovalene structures were generated using the CHARMM36 force field through the Ligand Reader and Modeler tool, available online at CHARMM-GUI (http://www.charmm-gui.org/?doc=input/ligandrm) [37]. The entire system was subsequently solvated in an orthorhombic water box using TIP3P water models, with 0.15 M concentrations of Na^+^ and Cl^−^ ions added to maintain electrostatic neutrality in a solvent buffer box measuring 25 Å³. Energy minimization was performed on the system, followed by equilibration for a duration of 10 ns. The molecular dynamics trajectories obtained from these simulations were analyzed and visualized using VMD 1.9.4 software.

Principal component analysis (PCA) was performed on the covariance matrix of atomic positional fluctuations after least-squares fitting of the MD trajectories. The first two principal components (PC1 and PC2), which capture the largest and second-largest variances in the system’s motions, were used as indicators to describe essential dynamics and to construct the conformational free energy landscape. PC1 reflects the dominant collective motion, while PC2 captures the next most significant orthogonal motion.

### Statistical analysis

Data are presented as the mean values ± standard deviations from three separate experiments. The disparities between the controls and tests were examined using analysis of variance. One-way or two-way ANOVA was applied as appropriate for each dataset via GraphPad Prism version 6 software. Statistical significance is attributed to findings at *P* < 0.05. Degrees of freedom for each comparison are indicated in the corresponding results section.

## Results

### Assessment of antimicrobial activity, MIC values, sub-MIC, growth monitoring, and viable bacterial count

Antimicrobial properties of Rottlerin and Ciprofloxacin (which was used as a positive control in this study) were evaluated against *K. pneumoniae* (ATCC700603). Zones of clearance were then measured (Supplementary Table S2). *K. pneumoniae* was resistant to Rottlerin but showed sensitivity to Ciprofloxacin. The MICs, determined according to CLSI (2020), were 1.5 mg/mL for Rottlerin and 0.002 mg/mL for Ciprofloxacin (Table 2).

**Table 2:** Minimum inhibitory concentration (MIC) values of Rottlerin and ciprofloxacin against *K. pneumoniae* (ATCC700603)

Compound	MIC (mg/mL)
Rottlerin	1.5
Ciprofloxacin	0.002

To further evaluate whether sub-MIC levels of Rottlerin affect bacterial growth, we monitored the OD600 values of *K. pneumoniae* cultures over 24 h in the presence of 0.375 mg/mL of Rottlerin (¼ MIC) and 0.001 mg/mL of Ciprofloxacin (½ MIC). The OD values of the Rottlerin-treated group increased over time, confirming bacterial growth (Supplementary Figure S3). However, they remained lower than the untreated control, which we attribute to a reduction in cell size rather than inhibition in growth. These findings were further supported by TEM images (Fig. 3) and by viable count assays (Supplementary Table S4), which confirmed that bacterial viability was unaffected. Collectively, these results indicate that while Rottlerin reduces capsule size and alters cell dimensions, it does not inhibit bacterial growth or viability at sub-MIC levels.

### Inhibitory effects of Rottlerin on biofilm assembly

**Fig. 2:**
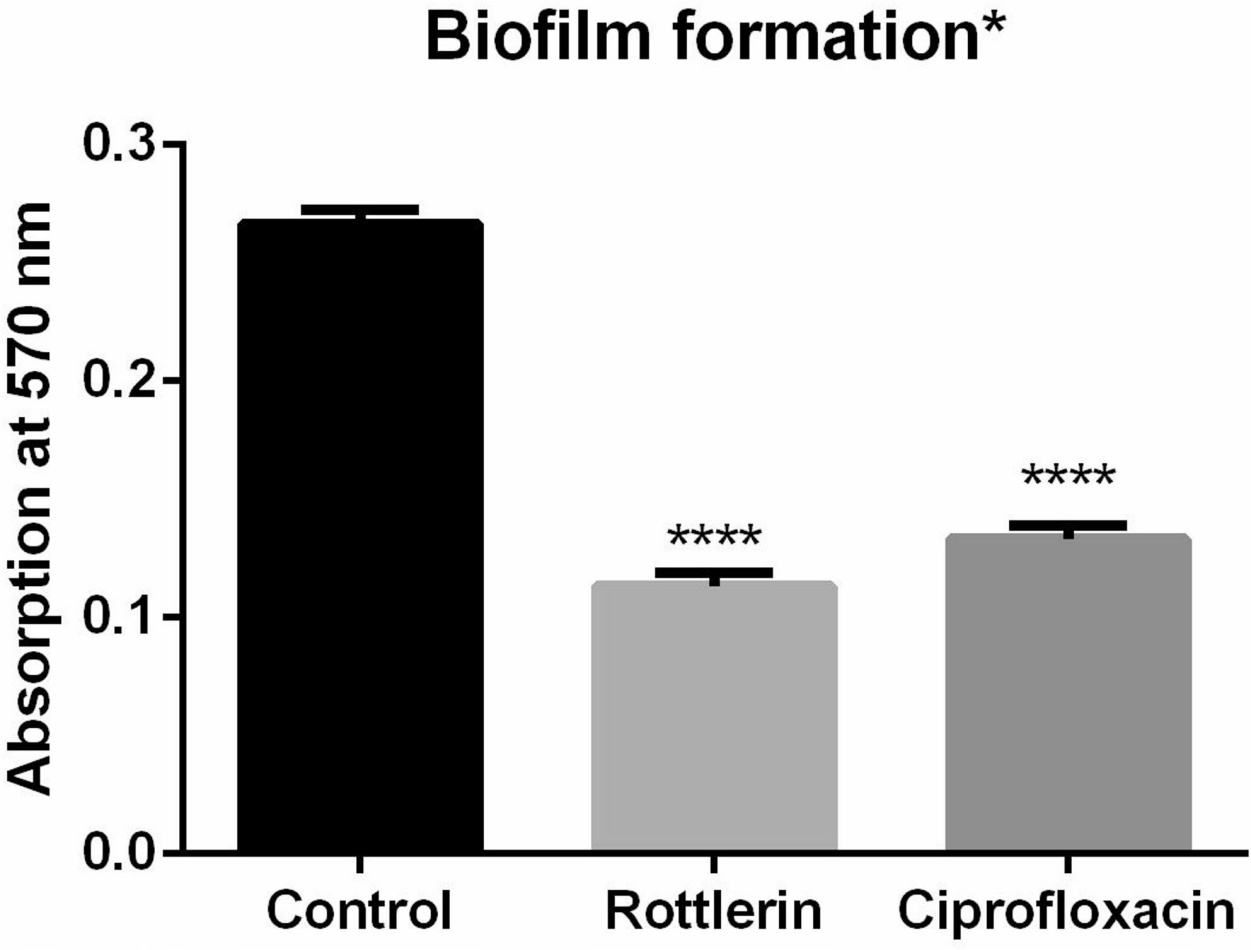
Inhibition of biofilm formation of Rottlerin and Ciprofloxacin. *Data represent the average of three replicates, expressed as mean ± SD of the absorbance measured at 570 nm. Statistical significance relative to the untreated control was evaluated at *P* < 0.0001. Data were analyzed using one-way ANOVA (F (2,6) = 625.3), performed in GraphPad Prism version 6

A crystal violet experiment was utilized to determine the inhibiting effect of sub-MIC Rottlerin (0.375 mg/mL, ¼ MIC) and Ciprofloxacin (0.001 mg/mL, ½ MIC). This test resulted in 57.6% and 50% inhibitions, respectively, in biofilm production, as shown in Fig. 2.

### Changes in bacterial capsule size

**Fig. 3:**
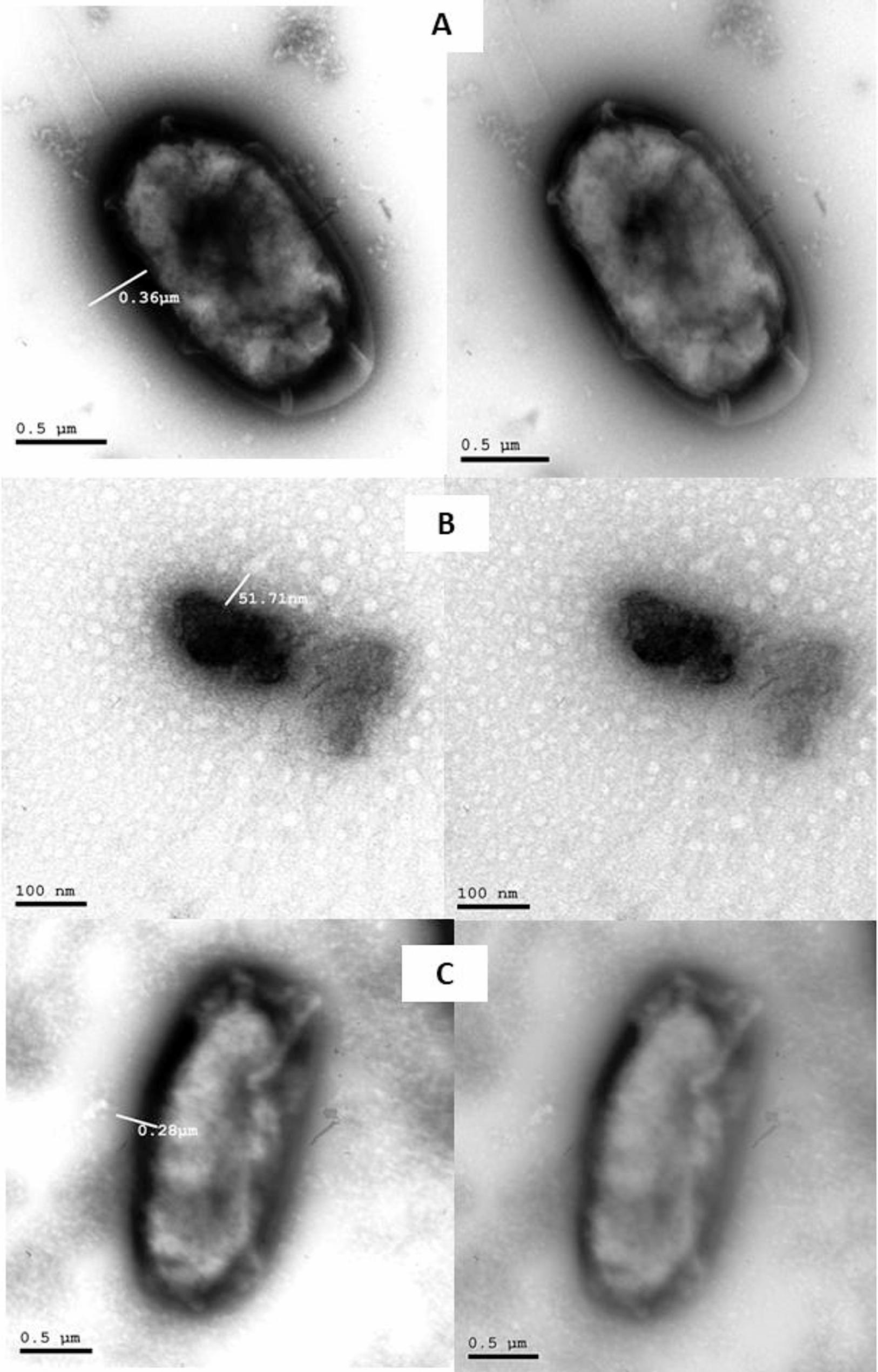
TEM showing changes in the capsule size of *K. pneumoniae.* (**A)** *K. pneumoniae* without treatment (scale bar: 0.5 μm); (**B**) *K. pneumoniae* after Rottlerin at sub-MIC treatment (scale bar: 100 nm); (**C**) *K. pneumoniae* after Ciprofloxacin at sub-MIC (scale bar: 0.5 μm). Different scale bars reflect the magnification required for optimal visualization of each sample. Representative images are shown. Lines mark the capsules. Capsule thickness was quantified using Gatan Digital Micrograph software

Transmission Electron Microscopy (TEM) was used to determine the capsule size. Representative electron micrographs of samples are shown in Fig. 3A-C. Capsule thickness was measured from micrographs using Gatan Digital Micrograph software (Gatan, Inc., Pleasanton, CA, USA). Measurements were taken from calibrated images to ensure accuracy. *K. pneumoniae* (ATCC700603) had a capsule thickness of 360 nm. The ability of Rottlerin and Ciprofloxacin to reduce capsule size was examined. As shown in Fig. 3B, Rottlerin exerted a significant effect on the capsule size. There was an 85.6% decrease in capsule size in the presence of Rottlerin, while a 33.3% decrease was recorded in the presence of Ciprofloxacin (Fig. 3C) compared to the untreated control (Fig. 3A). The morphological difference in Fig. 3B compared to the other panels is due to the different scale used in TEM imaging (scale bar = 100 nm in Fig. 3B vs. 0.5 μm in Fig. 3A and C). Despite the reduction in bacterial size as shown in Fig. 3B, tracking bacterial growth (Figure S3) and viable count (Table S4) showed that Rottlerin did not suppress bacterial proliferation, and the bacteria still grew normally. However, OD600 values after 24 h were decreased compared to the control, indicating a decrease in turbidity, which confirms a reduction in bacterial size.

### The impact of Rottlerin and Ciprofloxacin on the expression of virulence genes

**Fig. 4:**
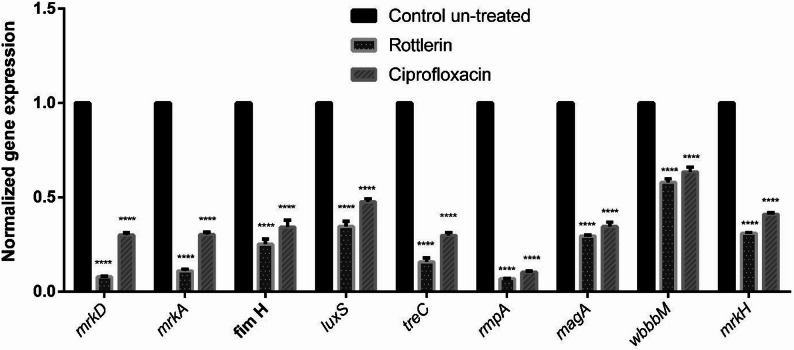
Downregulation of *mrkD, mrkA, fimH, luxS, treC, rmpA, magA, wbbM, *and *mrkH* expression in the presence of Rottlerin and Ciprofloxacin. Data represent the average of three replicates. The results are shown as the mean ± SD of the normalized gene expression. The *K. pneumoniae 16–23 S ITS* gene was used as an internal standard, and statistical significance was assessed against the untreated control at *P* < 0.0001. Two-way ANOVA indicated a significant effect of treatment (F (2, 54) = 21191, *P* < 0.0001)

The influence of Rottlerin (0.375 mg/mL, ¼ MIC) and Ciprofloxacin (0.001 mg/mL, ½ MIC) on the expression of virulence genes was examined using fluorescence real-time PCR. As shown in Fig. 4, Rottlerin at sub-MIC downregulated *mrkD*, *mrkA*, *fimH*,* luxS*,* treC*,* rmpA*,* magA*,* wbbM*, and *mrkH* expression by 92.12%, 89%, 74.91%, 65.49%, 84.23%, 93.14%, 70.58%, 41.96%, and 69.01%, respectively (*P* < 0.05). Ciprofloxacin at sub-MIC downregulated (*P* < 0.05) the same virulence gene expressions by 69.96%, 69.75%, 65.97%, 52.37%, 70.17%, 89.56%, 65.49%, 36.49%, and 58.96%, respectively.

### Docking-based virtual screening

#### MrkH as a probable target for Rottlerin

As an initial step in elucidating the potential molecular target responsible for the antibiofilm activity of Rottlerin against *K. pneumoniae*, we employed the PharmMapper platform to perform a reverse pharmacophore-based virtual screening. This strategy was selected to generate a rational hypothesis regarding the most probable protein targets for Rottlerin during its mechanism, particularly those implicated in biofilm formation. PharmMapper operates by aligning the pharmacophoric features of a query molecule against a curated database of receptor-based pharmacophore models derived from proteins with experimentally solved structures deposited in the Protein Data Bank (PDB). The platform assigns a “fit score” to each target, reflecting the degree of compatibility between the small molecule and the pharmacophore model of the target’s ligand-binding site [26].

**Fig. 5:**
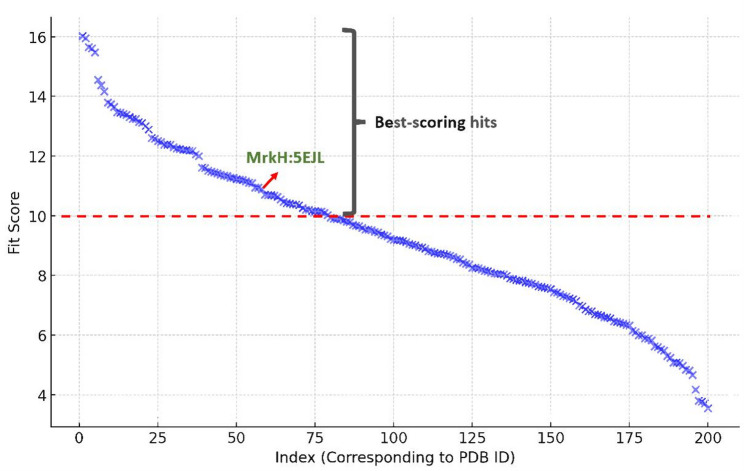
PharmMapper-based reverse pharmacophore mapping results for Rottlerin, illustrating the ranked fit scores of potential protein targets from the Protein Data Bank (PDB). Each blue cross corresponds to a target structure, plotted in descending order of fit score. The red dashed line indicates the general significance threshold (fit score = 10). *MrkH* (PDB ID: 5EJL), a c-di-GMP-dependent transcriptional activator involved in *K. pneumoniae* biofilm formation, is marked with a red arrow. Its placement among the top 50% of hits suggests a potential interaction with Rottlerin, supporting its selection for further docking and molecular dynamics-based validation as a candidate antibiofilm target

The resulting fit scores were plotted in descending order, as shown in Fig. 5. Each blue cross represents a protein structure ranked by its fit score with Rottlerin, as shown in Table S5. A red dashed line at the score of 10 demarcates a general threshold above which targets are considered to have meaningful pharmacophoric compatibility. Notably, MrkH (PDB ID: 5EJL), a transcriptional activator that governs the expression of type 3 fimbriae genes and plays a pivotal role in *K. pneumoniae* biofilm formation, was identified among the top 50% of hits (Fit score = 10.89). This suggests that Rottlerin may exert its antibiofilm effect through interaction with MrkH, potentially disrupting its ability to bind c-di-GMP or regulate fimbrial gene expression [29].

Given the relevance of MrkH in mediating surface adherence and biofilm maturation, its identification as a high-ranking target provided a compelling rationale for further investigation. As a next step, we validated this in silico prediction through a focused molecular docking analysis, wherein Rottlerin was redocked into the active site of MrkH to explore the structural and energetic basis of the interaction. This was followed by molecular dynamics (MD) simulations to assess the stability of the complex over time and the computation of the absolute binding free energy, thereby providing more concrete insights into the binding affinity and the potential of Rottlerin as a MrkH-targeting antibiofilm agent.

#### Docking and MD simulation

The comparative structural analysis of the MrkH binding pocket reveals striking differences between the native ligand c-di-GMP and the potential inhibitor Rottlerin, both in terms of interaction patterns and the spatial organization of key binding residues.

**Fig. 6:**
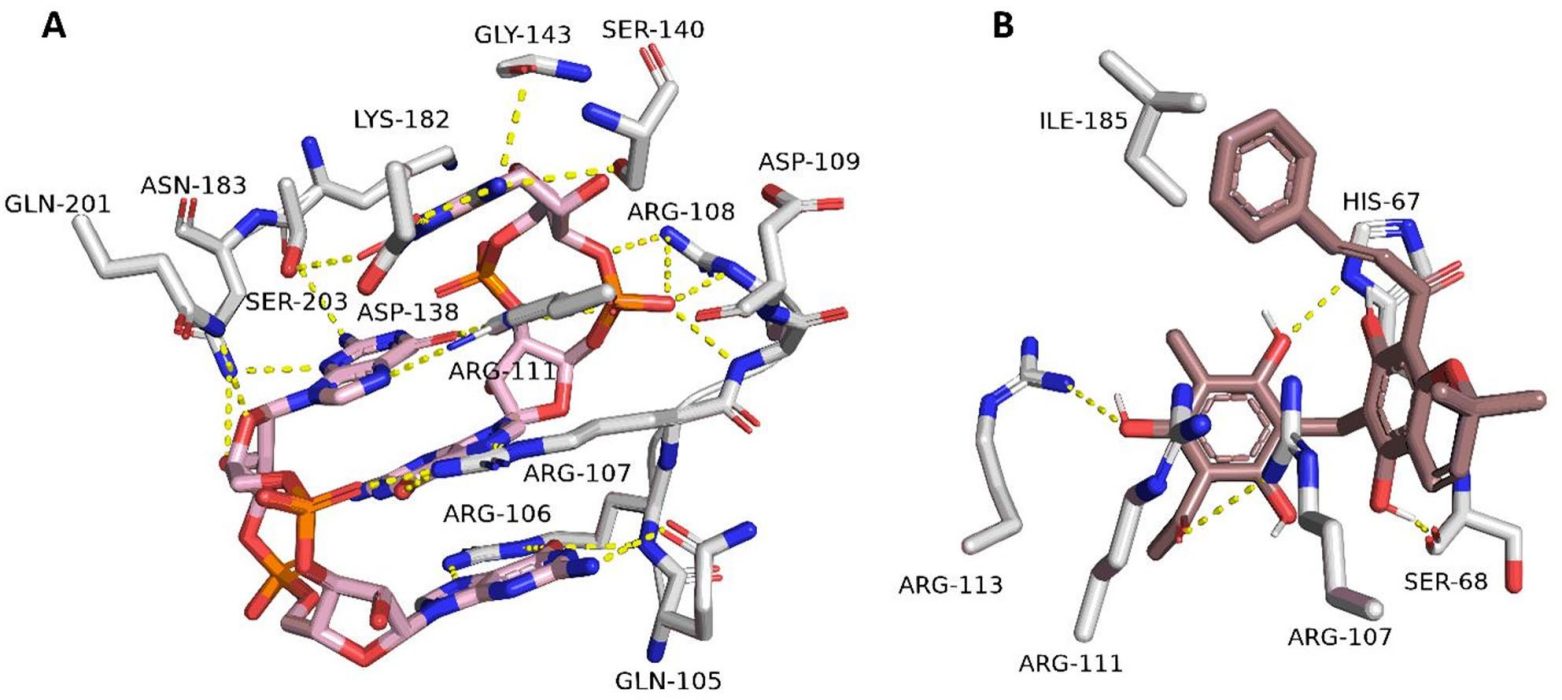
Binding interactions of MrkH with c-di-GMP (**A**) and Rottlerin (**B**). Panels illustrate the modeled interaction patterns of the native ligand (c-di-GMP) and Rottlerin within the MrkH binding region, providing a comparative visual representation of their binding modes

Figure 6A illustrates the co-crystallized conformation of the c-di-GMP dimer within MrkH’s PilZ domain, forming an intricate network of interactions that indicate its high-affinity binding and allosteric activation. Two mutually intercalated c-di-GMP molecules are held in place by a dense array of hydrogen bonds and electrostatic interactions involving conserved residues such as Arg107, Arg111, Arg108, Arg106, Asp109, Asp138, Ser203, and Gln105. Of functional significance are the cation-π interactions formed by Arg107 and Arg111 with the guanine bases of the c-di-GMP dimer, a recognition motif conserved among PilZ domain effectors and crucial for locking MrkH into its active DNA-binding conformation. The guanine stacking interaction between the two cyclic nucleotides further stabilizes the complex, augmenting its rigidity [29]. The importance of these contacts is underscored by mutagenesis studies; the substitution of Arg111 leads to a near-total loss of c-di-GMP binding affinity, and Arg107 mutations result in a 15-fold reduction in binding, confirming their critical role in ligand recognition and stabilization.

On the other hand, Fig. 6B presents the predicted docking pose of Rottlerin within the same binding pocket. Despite lacking the cyclic nucleotide scaffold of c-di-GMP, Rottlerin aligns favorably, forming an alternative interaction network that partially overlaps with the native ligand’s binding site. Notably, Arg107 engages in two distinct cation-π interactions with the aromatic rings of Rottlerin, mimicking its guanine-stacking interactions with c-di-GMP. Additional stabilizing hydrogen bonds are observed with Ser68, His67, Arg111, Arg113, and Ile185 residues that anchor the ligand into the pocket and prevent rapid egress. However, unlike the native complex, the hydrogen bonding network in the Rottlerin-bound pose is more localized and does not engage the extended polar surface of the PilZ domain to the same degree. The lack of intercalated stacking and reduced hydrogen bonding density suggests that while Rottlerin fits well within the binding site, it does not enforce the same conformational constraints as c-di-GMP.

In summary, these findings suggest that Rottlerin effectively competes for the c-di-GMP binding pocket in MrkH by engaging key residues—particularly Arg107—through pi-cation and hydrogen bond interactions. However, it likely fails to induce the extended structural locking and domain rearrangements necessary for functional activation.

To further validate and analyze these interactions, we subjected both structures to a 600 ns-long MD simulation. At first, we analyzed the binding stability of each molecule inside MrkH’s binding site in terms of their root mean square deviations (RMSD).

**Fig. 7:**
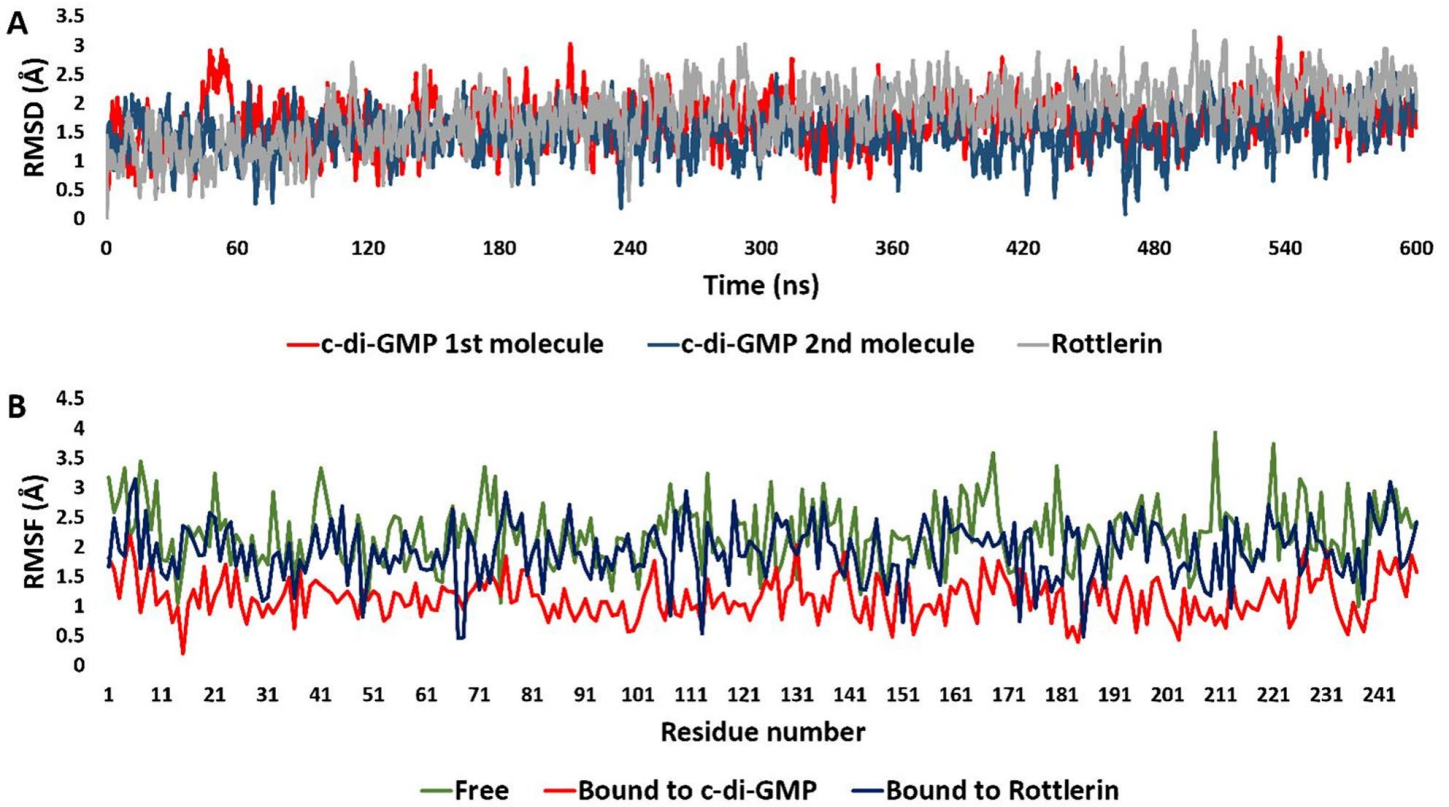
**A** Root mean square deviation (RMSD) trajectories of two intercalated c-di-GMP molecules (red and navy) and Rottlerin (gray) within the *MrkH* binding pocket over a 600-ns simulation. **B** Root mean square fluctuation (RMSF) profiles of the *MrkH* protein in its free form (green), bound to c-di-GMP (red), and bound to Rottlerin (navy blue), illustrating ligand-induced effects on protein flexibility across 600 ns of molecular dynamics simulation

The RMSD trajectories presented in Fig. 7A reflect the time-dependent positional stability of two c-di-GMP molecules and Rottlerin when bound within the MrkH binding pocket over a 600 ns simulation. Both c-di-GMP molecules—shown in red and navy—exhibit initial fluctuations during the early stages of the simulation (first ~ 50 ns), likely corresponding to minor conformational accommodation or settling into the binding cleft. Beyond this early adaptation phase, both molecules stabilize with RMSD values vastly fluctuating within the 1.0 to 2.0 Å range, punctuated by intermittent spikes that remain transient and rapidly dampened. These patterns suggest that the c-di-GMP dimer maintains a relatively consistent orientation and binding pose within the pocket, in line with its established role as a natural and tightly bound activator of MrkH.

Notably, the two c-di-GMP molecules remain interlocked and in close register throughout the simulation, reinforcing previous crystallographic findings that highlight their sandwich-like stacking geometry, stabilized by π–π interactions and flanked by critical polar contacts with MrkH residues. The overall low RMSD values, along with the recovery from transient displacement events, support the interpretation that these interactions are resilient and maintain the functional ligand-binding architecture.

Rottlerin, represented by the gray trace, follows a similar overall pattern. After an initial phase of positional adjustment, its RMSD values stabilize in a range that overlaps significantly with those of the c-di-GMP molecules. It consistently fluctuates between approximately 1.3 and 2.5 Å throughout the simulation, without any signs of drift, dissociation, or significant conformational instability. This indicates that Rottlerin remains securely positioned within the binding cleft of MrkH over time and does not behave as a weakly bound or transient ligand. In fact, its dynamic profile is remarkably comparable to that of c-di-GMP, suggesting that it engages the binding site with similar positional fidelity.

A more nuanced view reveals that while the amplitude of Rottlerin’s fluctuations is within the same range, its RMSD trace is slightly more irregular—lacking the flatter, more stable plateaus occasionally observed in the c-di-GMP trajectories. This could reflect the absence of a secondary stacking partner (as c-di-GMP binds as a sandwiched dimer) or fewer anchoring contacts. Rottlerin likely explores minor local rearrangements within the pocket, sufficient to maintain occupancy but potentially inadequate to induce the conformational tightening needed for MrkH activation.

Taken together, these RMSD patterns strongly support the notion that Rottlerin behaves as a structurally stable binder of MrkH, with a dynamic signature that closely mirrors that of the natural activator c-di-GMP. However, the subtle differences in binding rigidity and the absence of known activating interactions suggest that Rottlerin, while stably bound, likely acts as a non-activating mimic—capable of occupying the same physical space without triggering the allosteric changes required for downstream transcriptional regulation. This interpretation reinforces the potential of Rottlerin as a competitive inhibitor that stabilizes the protein-ligand complex without functionally activating it.

The root mean square fluctuation (RMSF) profiles **(**Fig. 7B**)** offer an additional view of how MrkH responds to different molecular environments, revealing its structural flexibility and the stabilizing influence of its ligands. When unbound, MrkH displays marked fluctuations across its sequence—most notably at the N- and C-termini and within several internal loop regions. These areas, which inherently lack rigid secondary structure, move more freely, reflecting the protein’s dynamic nature in its native, inactive state. This flexibility likely facilitates ligand accommodation but also suggests that in the absence of a stabilizing factor, MrkH remains conformationally loose and functionally inert.

Upon binding to its physiological effector, c-di-GMP, the protein adopts a noticeably more restrained dynamic profile. Across nearly the entire sequence, the RMSF values drop, indicating a global stabilization effect. This is especially pronounced at the functionally critical PilZ domain, where residues like Arg107, Arg111, and Ser203—central to c-di-GMP recognition—become significantly less mobile. This rigidity signals the locking in of a functional conformation, ready for DNA binding and transcriptional activation. It is clear that c-di-GMP doesn’t just bind—it structurally organizes MrkH into its biologically active form.

In contrast, the Rottlerin-bound form of MrkH tells a different story. While Rottlerin does appear to engage the protein—inducing localized stabilization at residues such as Arg107, Arg113, His67, Ser68, and Ile185—it doesn’t reproduce the full stabilizing effect seen with c-di-GMP. The rest of the protein, particularly in loop-rich and terminal regions, remains more flexible. This partial stabilization suggests that while Rottlerin occupies the binding pocket and forms some meaningful interactions, it does not drive the conformational change required for functional activation. In this context, Rottlerin may act more like a competitive inhibitor—binding without triggering activity, perhaps even locking MrkH in an inactive pose.

When overlaid with MrkH’s secondary structure, the RMSF data also highlight how different structural elements behave under these conditions. As expected, β-sheet regions—prevalent in MrkH according to the crystallographic data (PDB ID: 5Y6G)—remain relatively stable regardless of the ligand. These β-sheets likely form the rigid core of the protein. In contrast, loops and α-helices show more pronounced differences, adapting dynamically depending on whether the protein is ligand-free, bound to c-di-GMP, or complexed with Rottlerin.

Together, these observations reinforce the idea that ligand binding is not merely a binary event, but a structural negotiation—one that determines whether MrkH will remain idle or transition into its active, gene-regulating form.

#### Principal component analysis (PCA)

Principal component analysis (PCA) was employed to interrogate the conformational space sampled by the MrkH protein under three distinct molecular conditions: ligand-free (apo), bound to its natural effector c-di-GMP, and bound to the small-molecule Rottlerin. The resulting projection, derived from the simulated molecular dynamics (MD) trajectories, offered a detailed and visually coherent depiction of how each binding state reshapes the protein’s structural landscape.

**Fig. 8:**
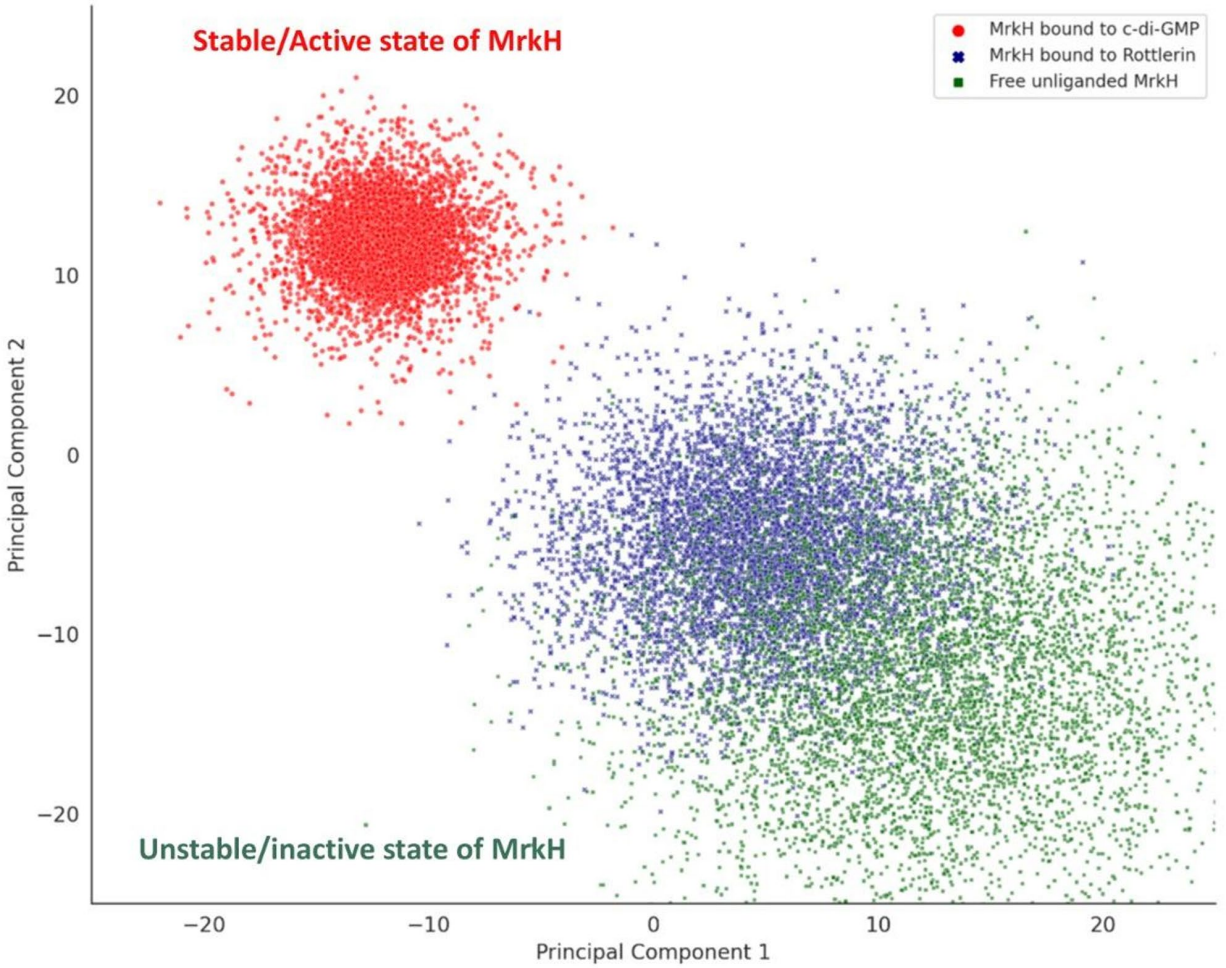
Principal Component Analysis (PCA) of the molecular dynamics trajectories of *MrkH* under three ligand-binding conditions: free (unliganded), bound to Rottlerin, and bound to c-di-GMP. Each point represents a protein conformation sampled during simulation and projected into a two-dimensional PCA space based on backbone atomic fluctuations. The c-di-GMP-bound *MrkH* conformers (red) form a compact, well-confined cluster in the upper-left quadrant, reflecting a structurally stabilized and activation-competent state. In contrast, the free protein (dark green) occupies a broad and diffuse region in the lower-right quadrant, indicative of high conformational plasticity in the absence of ligand. Rottlerin-bound *MrkH* (dark blue) forms a moderately spread cluster situated between the c-di-GMP and apo states, with partial overlap toward the unliganded ensemble

Figure 8 showed that the c-di-GMP–bound form of MrkH occupies a compact and well-confined cluster in the upper-left quadrant of the PCA plot, centered around principal component coordinates (PC1 ≈ − 12, PC2 ≈ + 12). This tight spatial grouping reflects the stabilizing influence of c-di-GMP, which constrains the protein into a limited range of conformational states. Such structural restraint is consistent with the known functional role of c-di-GMP in activating MrkH, facilitating its interaction with target DNA through allosteric rigidification. Notably, the c-di-GMP ensemble exhibited a dense core of points with a faintly dispersed surrounding shell, capturing both the structural precision of the activation-ready state and the minor conformational breathing permitted within the binding pocket.

By contrast, the Rottlerin-bound MrkH ensemble displayed a moderately dispersed cluster, distinctly separated from the c-di-GMP configuration and centered around PC1 ≈ + 5, PC2 ≈ − 5. While the distribution remained narrower than that of the free form, it was appreciably broader than the c-di-GMP ensemble, suggesting that Rottlerin binding confers only partial stabilization. This intermediate behavior is indicative of a binding event that anchors select contact residues but does not elicit the coordinated conformational reorganization characteristic of full activation. The displacement of the Rottlerin cluster away from the c-di-GMP space further supports the interpretation that Rottlerin does not mimic the active-state geometry of MrkH.

The free protein, in its apo form, occupied a broadly scattered region in the lower-right quadrant (PC1 ≈ + 12, PC2 ≈ − 12). This diffuse pattern is emblematic of a conformationally promiscuous state, with the protein sampling a wide array of geometries in the absence of a bound ligand. The overlap between the Rottlerin and apo clusters, though limited, suggests that Rottlerin maintains MrkH in a dynamic regime that resembles the unbound state more than the c-di-GMP-bound form.

Altogether, the PCA analysis reinforces the distinct conformational consequences of each binding condition. While c-di-GMP locks MrkH into a stable, functionally poised configuration, Rottlerin appears to stabilize the protein only partially, maintaining it in a non-activated, yet non-random, conformational state. This intermediate behavior supports the proposed role of Rottlerin as a competitive but non-activating ligand—occupying the c-di-GMP site without inducing the structural transitions necessary for transcriptional activation. The apo state, by contrast, remains dynamically unconstrained, underscoring the allosteric control exerted by ligand binding. These findings provide a structural basis for understanding the functional divergence between ligand classes and establish a mechanistic framework for evaluating MrkH-targeted antibiofilm agents.

## Discussion

The rising threat of multidrug-resistant *Klebsiella* infections has become a significant concern in healthcare settings, with *K. pneumoniae* being particularly notorious for its capacity to develop resistance mechanisms, including extended-spectrum β-lactamases (ESBLs) and carbapenemases [38]. As a result, patients with *Klebsiella* infections have limited options for treatment, leading to more extended hospital stays, higher mortality rates, and a greater risk of outbreaks [39]. The urgent need for novel therapeutic strategies is highlighted by the declining efficacy of conventional antibiotics [40]. Therefore, novel approaches, such as targeting virulence factors instead of bacterial viability, must be studied to combat this enemy.

Recent studies have demonstrated that numerous compounds exhibit anti-virulence activity. For example, fructus mume reduced mucoviscosity and the production of capsular polysaccharide (CPS) in *K. pneumoniae*. Additionally, citric acid, an abundant organic acid in fructus mume extracts, exhibited an inhibitory effect on the growth and CPS production of *K. pneumoniae* [10]. Similarly, an in vitro study reported that tea polyphenols inhibited the production of exopolysaccharides and the formation of biofilms in *K. pneumoniae* at sub-MIC [11]. In addition, one study demonstrated that Gallic acid hinders the growth of hypervirulent *K. pneumoniae* and also suppresses CPS production in the bacterium [41]. Furthermore, a separate study has shown that essential oils, including thyme oil and tea tree oil, effectively down-regulated the expression of capsule-related genes *rmpA* and *magA*, indicating their antagonistic capabilities against *Klebsiella.* Reducing *Klebsiella* virulence by the combined action of essential oils and antibiotics would therefore diminish the pathogen’s capacity to induce infection [42].

Among the key virulence-related targets, *Mr*kH is designated as a “biofilm switch” because its activation triggers a cascade of processes that facilitate biofilm formation. It acts as a transcriptional activator of the *mrk* gene cluster, specifically regulating *mrkHI* expression. Upon activation by c-di-GMP, MrkH undergoes structural modifications that facilitate its binding to the area upstream of the *mrkA* promoter. This binding encourages the transcription of the *mrkABCDF* operon, which is essential for making type 3 fimbriae required for biofilm formation [43].

Given its crucial role in biofilm formation and gene regulation, targeting MrkH with anti-virulence agents is a promising strategy to prevent antibiotic resistance by disrupting biofilm formation and combating chronic *K. pneumoniae* infections.

This study explores Rottlerin’s anti-virulence potential, thereby contributing to the development of innovative and sustainable therapeutic approaches. We examined the role of Rottlerin, a natural compound derived from the plant *Mallotus Philippensis*, also called the Kamala Tree, as a potential anti-virulence agent. Supporting this premise, several studies have demonstrated Rottlerin’s antimicrobial and antibiofilm activities. A study by Silva et al. states that Rottlerin exhibited antifungal and antibiofilm activities against certain yeast strains [44]. Moreover, according to a recent study, the Rottlerin scaffold shows great promise in inhibiting *Pseudomonas aeruginosa*’s quorum-sensing mechanism. More specifically, Rottlerin derivatives showed potent quorum-sensing inhibitory activity, moderate biofilm inhibitory efficacy, and reduced pyocyanin synthesis [45].

The molecular and dynamic characterization of MrkH presented here offers a coherent narrative that links ligand-specific binding interactions to global conformational behavior, with direct implications for understanding MrkH regulation and for the rational design of antibiofilm agents. At the center of this analysis lies the contrasting behavior of MrkH when bound to its natural effector, c-di-GMP, versus its interaction with the small-molecule Rottlerin. The differences, while subtle at the atomic scale, manifest clearly across structural, dynamic, and energetic dimensions.

The co-crystallized c-di-GMP dimer binds MrkH with remarkable precision, stabilized by a network of polar contacts and signature π-cation interactions involving Arg107 and Arg111. These interactions anchor the guanine bases of the intercalated cyclic nucleotides, forming a tight scaffold that restrains the protein’s conformational mobility and effectively locks the PilZ domain into a state competent for DNA binding. RMSF analysis of the c-di-GMP–bound complex confirms this rigidification, with markedly lower atomic fluctuations across the entire sequence, including loop regions and the structurally dominant β-sheets. This is further reinforced by the PCA of the MD trajectories, where c-di-GMP–bound conformers form a dense, isolated cluster—a conformational fingerprint of an allosterically activated state.

By contrast, Rottlerin binds in a spatially similar region of the PilZ domain but engages the protein through a distinct set of interactions. While lacking the cyclic and nucleotide-based features of c-di-GMP, Rottlerin nonetheless exploits key residues,most notably Arg107, which forms two π-cation interactions with the molecule’s aromatic rings. These contacts mimic the anchoring role of guanine but do not translate into the same level of structural organization. The remaining interactions, including those with Arg113, His67, Ser68, and Ile185, are stabilizing but confined, failing to extend into the polar surface network that underpins complete conformational reprogramming in the c-di-GMP complex.

These atomic distinctions are reflected in the dynamic signatures. RMSF analysis shows that Rottlerin-bound MrkH exhibits moderate stabilization at contact points yet retains flexibility across loop regions and termini. The PCA trajectory confirms this intermediate behavior: the Rottlerin**-**bound state forms a moderately dispersed cluster, clearly separated from the c-di-GMP ensemble but still distinguishable from the broader and more chaotic distribution of the unliganded form. This separation implies that Rottlerin imposes some local order—likely enough to outcompete c-di-GMP for binding—but not the global locking needed to enable transcriptional activation.

Taken together, these data support a model in which Rottlerin functions as a non-activating competitive binder: it fits well into the c-di-GMP pocket, stabilizes key residues like Arg107 through π-cation interactions, and occupies the physical space required to block native ligand access. However, it does so without reproducing the allosteric communication required for functional activation. This distinction is critical. While binding affinity is necessary for inhibition, the inability to induce a rigid, c-di-GMP–like conformational state renders Rottlerin a silent occupant—effectively muting MrkH’s regulatory function without triggering its DNA-binding capacity.

These findings provide a mechanistic rationale for targeting MrkH with non-nucleotide inhibitors and reinforce the idea that c-di-GMP recognition involves not only binding but also structural orchestration. Rottlerin’s performance in this context offers a promising lead scaffold for further development, particularly if modifications can enhance domain-spanning interactions or increase rigidity at the ligand–protein interface. More broadly, this work offers a template for dissecting the interplay between binding geometry, protein flexibility, and functional output in c-di-GMP–dependent regulators.

The antimicrobial test and MIC assay results indicated that Rottlerin exhibited weak antimicrobial activity. Notably, the inhibition of biofilm and other virulence factors by Rottlerin seems to be independent of direct antimicrobial activity. The data further indicated that Rottlerin substantially impeded the biofilm development of *K. pneumoniae*. Treatment with Rottlerin at sub-MIC yielded a 57.6% reduction in biofilm development, whereas Ciprofloxacin at sub-MIC produced a 50% decrease relative to the untreated control. These data underscore the efficacy of both drugs in inhibiting biofilm formation, a critical virulence component that promotes bacterial persistence and resistance to antimicrobial treatments. Notably, the enhanced efficacy of Rottlerin indicates a more potent inhibitory mechanism, potentially via disruption of biofilm-related regulatory pathways, including those regulated by MrkH.

Considering the significant impact of biofilms on chronic infections and antibiotic resistance, these findings reinforce the advancement of anti-virulence techniques aimed at inhibiting biofilm formation to more effectively address *K. pneumoniae* infections.

Alongside biofilm inhibition, Rottlerin was also found to affect capsule production, another critical virulence determinant of *K. pneumoniae*. Considering the essential function of the capsule in immune evasion, surface adhesion, and biofilm protection, the apparent decrease in capsule size after treatment with Rottlerin supports its anticipated anti-virulence efficacy against *K. pneumoniae*. Transmission electron microscopy (TEM) examination demonstrated a significant reduction in capsule thickness, with Rottlerin at sub-MIC causing an 85.6% drop, whereas Ciprofloxacin at sub-MIC led to a 33.3% reduction compared to the untreated reference. These findings indicate that both agents undermine a principal defense mechanism of *K. pneumoniae*. Notably, the markedly enhanced impact of Rottlerin on capsule size suggests its substantial inhibitory capability, possibly through disruption of capsule production pathways. This underscores the idea that targeting biofilm-associated regulators, such as MrkH, could have wider ramifications, potentially hindering biofilm formation and diminishing capsule-mediated pathogenicity, hence increasing bacterial vulnerability to host immune responses and antimicrobial therapies.

To further understand the mechanism of biofilm and capsule inhibition, we examined the expression of key genes related to virulence. These genes include *mrkH*,* mrkD*,* mrkA*,* fimH*,* luxS*, *rmpA*,* magA*,* treC*,* and wbbM*, which are associated with the virulence of *K. pneumoniae*. Our findings emphasize the substantial influence of Rottlerin on key biofilm-related genes in *K. pneumoniae*, especially those governed by MrkH. At sub-MIC, Rottlerin significantly decreased *mrkH* expression by 69.01%, alongside considerable reductions in *mrkD* (92.12%), *mrkA* (89%), and *fimH* (74.91%), all of which are integral to fimbrial adhesion and biofilm structural integrity. Furthermore, genes associated with quorum sensing (*luxS*), lipopolysaccharide (LPS) biosynthesis (*wbbM*), and capsular polysaccharide (*rmpA*,* magA*,* treC*) were markedly downregulated. Ciprofloxacin exhibited significant gene downregulation, specifically targeting *mrkH*,* magA*, and *wbbM*, with decreases of 65.97%, 70.17%, and 89.56%, respectively.

Importantly, the in vitro gene expression data are consistent with our in silico model of Rottlerin as a non-activating competitive ligand of MrkH. At sub-MIC, Rottlerin markedly downregulated *mrkH* (69.01%), as well as *mrkA* (89%) and *fimH* (74.91%), which encode key structural components of type 3 fimbriae and type 1 fimbriae that are required for biofilm initiation and maturation. These transcriptional changes parallel the observed 57.6% reduction in biofilm biomass and the pronounced 85.6% decrease in capsule thickness, indicating that impaired fimbrial expression and altered surface architecture together underline the anti-virulence phenotype.

In line with these experimental findings, docking, MD, RMSF, and PCA analyses show that Rottlerin occupies the c-di-GMP binding pocket of MrkH via π-cation and hydrogen-bond interactions with critical residues such as Arg107 and Arg111, yet induces only partial stabilization of the PilZ domain and maintains an intermediate conformational flexibility between the apo and c-di-GMP–bound states.

Together, these data support a model in which Rottlerin competes with c-di-GMP for MrkH binding, preventing full allosteric activation and thereby dampening MrkH-dependent transcription of fimbrial and capsule-associated genes, which is directly reflected in the observed gene expression, biofilm, and capsule phenotypes.

Nonetheless, these findings are derived from in vitro and in silico analyses and therefore require future in vivo validation to confirm the biological relevance of Rottlerin’s anti-virulence effects under physiological conditions.

## Conclusion

Our study demonstrated that Rottlerin, at sub-MIC levels, reduces *K. pneumoniae* biofilms and capsule formation. Based on these findings, we hypothesize that Rottlerin has potential inhibitory effects on the production of virulence factors, capsule synthesis, and biofilm formation in *K. pneumoniae* through effects on MrkH. Our results show that the influence of MrkH goes beyond its basic role in biofilm formation, as the downregulation of *rmpA*,* magA*,* and wbbM* suggests a significant impact on capsule biosynthesis. This reveals that targeting MrkH could potentially disrupt capsule formation in addition to compromising biofilm integrity, thereby reducing *K. pneumoniae*’s virulence and resistance mechanisms. Although these findings point to a possible link between MrkH activity and a reduction in capsule size, further research is needed to clarify MrkH’s direct regulatory role in *K. pneumoniae* capsule biosynthesis pathways. Nonetheless, this study has constraints. The experiments were performed in vitro, and only a restricted set of virulence variables was evaluated. Additional in vivo experiments and more comprehensive mechanistic analysis are necessary to corroborate and enhance these findings.

Collectively, these findings highlight Rottlerin’s potential as a lead compound for the development of novel anti-virulence strategies.

## Supplementary Information

Supplementary Material 1. (Table S1)

Supplementary Material 2. (Table S2)

Supplementary Material 3. (Figure S3)

Supplementary Material 4. (Table S4)

Supplementary Material 5. (Table S5)

## Data Availability

All data generated or analysed during this study, including primer sequences for real-time PCR, are included in the manuscript and its supplementary information files. Structural data for the MrkH protein are available in the Protein Data Bank (PDB code: **5EJL)** [10.2210/pdb5EJL/pdb](https:/doi.org/10.2210/pdb5EJL/pdb).

## Abbreviations

*K*.: *Klebsiella *
*K. pneumoniae*: *Klebsiella pneumoniae *
MDR: Multidrug-resistant
WHO: World Health Organization
LPS: Lipopolysaccharide
MIC: Minimum inhibitory concentrations
Sub-MIC: Sub-minimum inhibitory concentration
TSB: Tryptone Soya Broth
CLSI: Clinical and laboratory standard institute
PBS: Phosphate-buffered saline
PTA: Phosphotungstic acid
PDB: Protein Data Bank
TEM: Transmission Electron Microscopy
MD: Molecular dynamics
RMSD: Root mean square deviations
RMSF: Root mean square fluctuation
PCA: Principal component analysis
PCR: Polymerase chain reaction
ESBLs: Extended-spectrum β-lactamases
CPS: Capsular polysaccharide
CFU: Colony Forming Unit
