# Supplementary material for: Mechanistic insights into Rottlerin’s inhibition of MrkH-mediated biofilm and capsule formation in Klebsiella pneumoniae
Source: BMC Microbiol. 2025 Dec 27;26:59. doi: 10.1186/s12866-025-04582-4 (PMC12849489; doi:10.1186/s12866-025-04582-4)
Supplement: Supplementary file 1 — Supplementary Material 1. (Table S1) [file 12866_2025_4582_MOESM1_ESM.docx]

**Mechanistic Insights into Rottlerin’s Inhibition of MrkH-Mediated Biofilm and Capsule Formation in *Klebsiella pneumoniae***

Rosette S. Hanna^1,2^*, Mohamed A. Sebak ^2^, Ahmed M. Sayed ^3,4^, Ahmed O. El-Gendy ^2^, Mostafa N. Taha ^1^

**1** Department of Microbiology and Immunology, Faculty of Pharmacy, Nahda University, Beni-Suef 62513, Egypt

**2** Department of Microbiology and Immunology, Faculty of Pharmacy, Beni-Suef University, Beni-Suef 62514, Egypt.

**3** Department of Pharmacognosy, Faculty of Pharmacy, Nahda University, Beni-Suef 62513, Egypt

4 Department of Pharmacognosy, college of Pharmacy, Almaaqal University, 61014 Basrah, Iraq

*Corresponding author: rosette.sameh@nub.edu.eg

**Table S1: Cycling conditions for SYBR green real time PCR**

| **Target gene** | **Reverse transcription** | **Primary**  **denaturation** | **Amplification (40 cycles)** | | | **Dissociation curve**  **(1 cycle)** | | |
| --- | --- | --- | --- | --- | --- | --- | --- | --- |
|  |  |  | **Secondary denaturation** | **Annealing**  **(Optics on)** | **Extension** | **Secondary denaturation** | **Annealing** | **^Final denaturation^** |
| *fimH* | 50˚C  30 min | 94˚C  15 min | 94˚C  15 sec. | 51˚C  30 sec. | 72˚C  30 sec. | 94˚C  1 min. | 51˚C  1 min. | 94˚C  1 min. |
| *mrkA* |  |  |  | 55˚C  30 sec. |  |  | 55˚C  1 min. |  |
| *mrkD* |  |  |  | 50˚C  30 sec. |  |  | 50˚C  1 min. |  |
| *luxS* |  |  |  | 55˚C  30 sec. |  |  | 55˚C  1 min. |  |
| *treC* |  |  |  | 52˚C  30 sec. |  |  | 52˚C  1 min. |  |
| *rmpA* |  |  |  | 52˚C  30 sec. |  |  | 52˚C  1 min. |  |
| *magA* |  |  |  | 51˚C  30 sec. |  |  | 51˚C  1 min. |  |
| *wbbM* |  |  |  | 55˚C  30 sec. |  |  | 55˚C  1 min. |  |
| *mrkH* |  |  |  | 51˚C  30 sec. |  |  | 51˚C  1 min. |  |
| *K. pneumoniae 16S-23S ITS* |  |  |  | 55˚C  30 sec. |  |  | 55˚C  1 min. |  |
