# Supplementary material for: Mechanistic insights into Rottlerin’s inhibition of MrkH-mediated biofilm and capsule formation in Klebsiella pneumoniae
Source: BMC Microbiol. 2025 Dec 27;26:59. doi: 10.1186/s12866-025-04582-4 (PMC12849489; doi:10.1186/s12866-025-04582-4)
Supplement: Supplementary file 2 — Supplementary Material 2. (Table S2) [file 12866_2025_4582_MOESM2_ESM.docx]

**Mechanistic Insights into Rottlerin’s Inhibition of MrkH-Mediated Biofilm and Capsule Formation in *Klebsiella pneumoniae***

Rosette S. Hanna^1,2^*, Mohamed A. Sebak ^2^, Ahmed M. Sayed ^3,4^, Ahmed O. El-Gendy ^2^, Mostafa N. Taha ^1^

**1** Department of Microbiology and Immunology, Faculty of Pharmacy, Nahda University, Beni-Suef 62513, Egypt

**2** Department of Microbiology and Immunology, Faculty of Pharmacy, Beni-Suef University, Beni-Suef 62514, Egypt.

**3** Department of Pharmacognosy, Faculty of Pharmacy, Nahda University, Beni-Suef 62513, Egypt

4 Department of Pharmacognosy, College of Pharmacy, Almaaqal University, 61014 Basrah, Iraq

*Corresponding author: rosette.sameh@nub.edu.eg

**Table S2. Antimicrobial activity of Rottlerin and Ciprofloxacin against *Klebsiella pneumoniae* (ATCC 700603). The zone of clearance was determined to assess susceptibility**

| Compound | Concentration (mg/mL) | Inhibition Zone in mm |
| --- | --- | --- |
| Rottlerin | 2.0 | 35 |
| Ciprofloxacin | 2.0 | 11 |
