# Supplementary material for: Mechanistic insights into Rottlerin’s inhibition of MrkH-mediated biofilm and capsule formation in Klebsiella pneumoniae
Source: BMC Microbiol. 2025 Dec 27;26:59. doi: 10.1186/s12866-025-04582-4 (PMC12849489; doi:10.1186/s12866-025-04582-4)
Supplement: Supplementary file 3 — Supplementary Material 3. (Figure S3) [file 12866_2025_4582_MOESM3_ESM.docx]

**Mechanistic Insights into Rottlerin’s Inhibition of MrkH-Mediated Biofilm and Capsule Formation in *Klebsiella pneumoniae***

Rosette S. Hanna^1,2^*, Mohamed A. Sebak ^2^, Ahmed M. Sayed ^3,4^, Ahmed O. El-Gendy ^2^, Mostafa N. Taha ^1^

**1** Department of Microbiology and Immunology, Faculty of Pharmacy, Nahda University, Beni-Suef 62513, Egypt

**2** Department of Microbiology and Immunology, Faculty of Pharmacy, Beni-Suef University, Beni-Suef 62514, Egypt.

**3** Department of Pharmacognosy, Faculty of Pharmacy, Nahda University, Beni-Suef 62513, Egypt

4 Department of Pharmacognosy, College of Pharmacy, Almaaqal University, 61014 Basrah, Iraq

*Corresponding author: [rosette.sameh@nub.edu.eg](mailto:rosette.sameh@nub.edu.eg)

**Table S4:** **Viable count assay of *K. pneumoniae* following treatment with sub-MIC levels of Rottlerin and Ciprofloxacin**.

Effect of sub-MIC Rottlerin on viable counts of *K. pneumoniae*. Cultures were grown for 24 h in the presence or absence of Rottlerin or positive control (Ciprofloxacin), then plated for CFU enumeration. “TNTC” indicates *too numerous to count*. “ND” indicates *not detected* (no colonies observed at this dilution).

| Dilution Factor | Control | | Ciprofloxacin | | Rottlerin | |
| --- | --- | --- | --- | --- | --- | --- |
|  | Colony Count | CFU/ml | Colony Count | CFU/ml | Colony Count | CFU/ml |
| 0.1 | TNTC | | TNTC | | TNTC | |
| 0.01 | TNTC | | TNTC | | TNTC | |
| 0.001 | TNTC | | TNTC | | TNTC | |
| 0.0001 | 720 | 7200000 | 680 | 6800000 | 840 | 8400000 |
| 0.00001 | 74 | 7400000 | 65 | 6500000 | 84 | 8400000 |
| 0.000001 | 7 | 7000000 | 6 | 6000000 | 6 | 6000000 |
| 0.0000001 | ND | | ND | | ND | |
| 0.00000001 | ND | | ND | | ND | |
| 0.000000001 | ND | | ND | | ND | |
| 0.0000000001 | ND | | ND | | ND | |
| 0.00000000001 | ND | | ND | | ND | |
| 0.000000000001 | ND | | ND | | ND | |
