# Supplementary material for: Mechanistic insights into Rottlerin’s inhibition of MrkH-mediated biofilm and capsule formation in Klebsiella pneumoniae
Source: BMC Microbiol. 2025 Dec 27;26:59. doi: 10.1186/s12866-025-04582-4 (PMC12849489; doi:10.1186/s12866-025-04582-4)
Supplement: Supplementary file 4 — Supplementary Material 4. (Table S4) [file 12866_2025_4582_MOESM4_ESM.docx]

**Mechanistic Insights into Rottlerin’s Inhibition of MrkH-Mediated Biofilm and Capsule Formation in *Klebsiella pneumoniae***

Rosette S. Hanna^1,2^*, Mohamed A. Sebak ^2^, Ahmed M. Sayed ^3,4^, Ahmed O. El-Gendy ^2^, Mostafa N. Taha ^1^

**1** Department of Microbiology and Immunology, Faculty of Pharmacy, Nahda University, Beni-Suef 62513, Egypt

**2** Department of Microbiology and Immunology, Faculty of Pharmacy, Beni-Suef University, Beni-Suef 62514, Egypt.

**3** Department of Pharmacognosy, Faculty of Pharmacy, Nahda University, Beni-Suef 62513, Egypt

4 Department of Pharmacognosy, College of Pharmacy, Almaaqal University, 61014 Basrah, Iraq

*Corresponding author: [rosette.sameh@nub.edu.eg](mailto:rosette.sameh@nub.edu.eg)

**Supplementary Figure S3.** Growth monitoring of *Klebsiella pneumoniae* (ATCC700603) over 24 h in the presence of sub-MIC of Rottlerin (¼ MIC) and Ciprofloxacin (½ MIC), compared with the untreated control. Optical density at 600 nm (OD₆₀₀) was measured at defined intervals. Data are presented as mean ± SD of three independent replicates.
